# PJI-TNM als neues Klassifikationssystem für Endoprotheseninfektionen: Eine Evaluation von 20 Fällen

**DOI:** 10.1007/s00132-020-03933-5

**Published:** 2020-06-12

**Authors:** Markus Rupp, Maximilian Kerschbaum, Viola Freigang, Susanne Bärtl, Florian Baumann, Andrej Trampuz, Volker Alt

**Affiliations:** 1 Klinik und Poliklinik für Unfallchirurgie, Universitätsklinikum Regensburg (UKR), Franz-Josef-Strauss-Allee 11, 93053 Regensburg, Deutschland; 2 Charité – Universitätsmedizin Berlin und Center for Musculoskeletal Surgery (CMSC), Berlin, Deutschland

**Keywords:** Biofilme, Gelenkersatz, Implantatinfektion, Retrospektive Studie, TNM-Klassifikation, Biofilms, Arthroplasty, Periprosthetic joint infections, Retrospective studies, TNM classification

## Abstract

**Hintergrund:**

Bisherige Klassifikationen für Protheseninfektionen beurteilen die Heterogenität der Infektion nur unzureichend. Die PJI-TNM-Klassifikation berücksichtigt auf Basis der onkologischen TNM-Klassifikation folgende entscheidende Kriterien bei Protheseninfektionen: Implantatart und -stabilität, Weichteilverhältnisse, Biofilmreife, Erregerart, Komorbiditäten des Patienten und Infektrezidive. Ziel dieser Arbeit ist es, die neue PJI-TNM-Klassifikation auf deren Anwendbarkeit in der klinischen Praxis zu überprüfen.

**Methoden:**

Im Rahmen einer Konzeptionsstudie wurde die PJI-TNM-Klassifikation bei 20 Patienten mit periprothetischen Schulter‑, Hüfte- oder Kniegelenksinfektion angewandt. Anhand der Patientenakte wurde die PJI-TNM-Klassifikation mit den übergeordneten Parametern T („tissue and implants“), N („non-eucaryotic cells and fungi“), M („morbidity“) und r („reinfection“), klassifiziert.

**Ergebnisse:**

Alle 20 Fälle (12 männlich, 8 weiblich, mittleres Alter 72,2 [40–88 Jahre]), darunter 13 Hüft-, 6 Knie- und eine Schulterprotheseninfektion, konnten mit der PJI-TNM-Klassifikation klassifiziert werden. Insgesamt zeigte sich eine große Heterogenität der Fälle: 12 Prothesen waren fest (T0), 6 gelockert (T1) und bei zwei Prothesen ein Weichteildefekt (T2) vorhanden. Bei 7 Prothesen wurde von unreifem Biofilm (N0) ausgegangen. 13 Prothesen wurden entsprechend eines reifen Biofilms klassifiziert (N1+N2). 9 Patienten waren nur geringgradig (M0), 7 Patienten mäßig (M1) und 3 Patienten schwer vorerkrankt (M2). Ein Patient lehnte die chirurgische Therapie ab (M3a). Bei 3 Fällen handelte es sich um eine Reinfektion (r).

**Schlussfolgerungen:**

Die aus der Onkologie stammenden Prinzipien der TNM-Klassifikation lassen sich auch bei periprothetischen Infektionen anwenden. Schon bei einer geringen Fallzahl ist eine deutliche Heterogenität periprothetischer Infektionen, wie sie auch im klinischen Alltag generell beobachtet wird, feststellbar. Diese wird durch die PJI-TNM-Klassifikation gut abgebildet und kann dadurch zukünftig eventuell zur Verbesserung bei der Therapieentscheidung beitragen.

## Abstract (en)

**Background:**

Current classifications for periprosthetic joint infections (PJIs) often lack a detailed description of the overall underlying situation of a patient. The PJI-TNM classification uses the principles of the TNM classification from oncology for the description of critical parameters in PJIs: affected joint, type of implant and implant stability, soft tissue conditions, maturity of biofilm formation, causative microorganism, comorbidities of the patient and recurrence of infection. The aim of the current work is to evaluate the feasibility of this new PJI-TNM classification in clinical practice.

**Methods:**

The PJI-TNM classification was used in 20 patients with hip, knee and shoulder PJIs. Based on a retrospective chart review, the respective parameters T (tissue and implants), N (non-eukaryotic cells and fungi), M (morbidity) and r (reinfection) were classified for each case.

**Results:**

All 20 cases (12 male, 8 female, average age 72.2 (40–88 years)) with 13 hip, 6 knee and 1 shoulder PJIs were to be classified with the new TNM-PJI classification system. There was a considerable heterogeneity among the cases: 12 protheses were fixed (T0), 6 were loosened (T1) and 2 were associated with a soft tissue defect (T2). Biofilm formation was considered immature in 7 cases (N0). Out of the PJIs, 13 were considered to be associated with mature biofilm formation. Out of the patients, 9 were systemically not or only mildly compromised (M0), 7 patients moderately (M1) and 3 patients (M2) severely compromised. One patient refused surgical treatment (M3a). Recurrent infections (r) were diagnosed in three cases.

**Conclusions:**

The principles of the TNM classification from oncology can also be used for the classification of PJIs. Despite the limited number of cases in this study, a considerable heterogeneity of the evaluated PJIs is shown, which is a phenomenon that is also known from clinical practice. This heterogeneity can be adequately addressed by this new classification, which might be beneficial in decision-making in the future.

## Hintergrund und Fragestellung

Eine periprothetische Infektion ist die häufigste Komplikation nach endoprothetischem Gelenkersatz des Knies und die dritthäufigste Komplikation nach Hüftgelenkersatz [15]. Mit zunehmender Alterung der Gesellschaft konnte in Deutschland, aber auch weltweit, eine Zunahme an Primärimplantationen von Hüft- und Kniegelenkendoprothesen verzeichnet und für die nächsten Jahre vorausgesagt werden [20, 25, 26]. Mit dieser Entwicklung geht auch ein absoluter Anstieg von Protheseninfektionen einher, der die behandelnden Ärzte, die betroffenen Patienten und deren soziales Umfeld vor eine immense Herausforderung stellt. Eine Protheseninfektion ist nicht nur mit längeren, sondern häufig auch mit mehrfachen Hospitalisierungen verbunden [14]. Neben einer Einschränkung der Lebensqualität und Mobilität sind Patienten einer deutlich erhöhten Mortalität ausgesetzt [11–13]. Nach einem Jahr wird die Mortalität nach Hüftprotheseninfektion mit 13,6 % und nach 5 Jahren mit 25,9 % beziffert [28], was mit der Mortalität verschiedener Tumorerkrankungen durchaus vergleichbar ist [4].

Der Ähnlichkeit zwischen periprothetischen Infektionen und Tumorerkrankungen wird durch den häufig verwendeten Satz „Behandele die Infektion wie einen Tumor“ Ausdruck verliehen. In der Onkologie ist die am weitest verbreitete Klassifikation die TNM-Klassifikation für maligne Tumoren. Die Grundlage der Klassifikation wurde in den späten 1940er- und frühen 1950er-Jahren durch Pierre Denoix gelegt. Die achte Version der onkologischen TNM-Klassifikation, die weltweit für die Mehrzahl maligner Tumoren Verwendung findet, wurde 2017 veröffentlicht [3]. Die TNM-Klassifikation basiert auf den wichtigsten Kriterien für die Prognose der Tumorerkrankung, wie der Ausbreitung des Tumors (T), des regionalen Lymphknotenbefalls (N) und der Metastasierung (M) des Tumors, welche je nach Ausdehnung zusätzliche Ziffern enthalten (z. B. T1–T4). Diese Klassifikation eines Tumors ermöglicht es, die bestmögliche Therapie für die Patienten abzuleiten.

**Figure 1:**
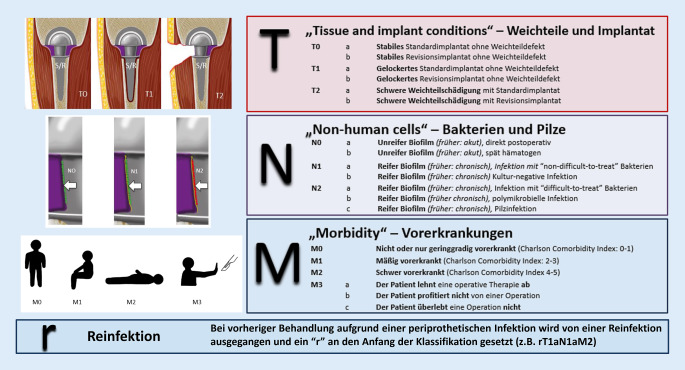

**Figure 2:**
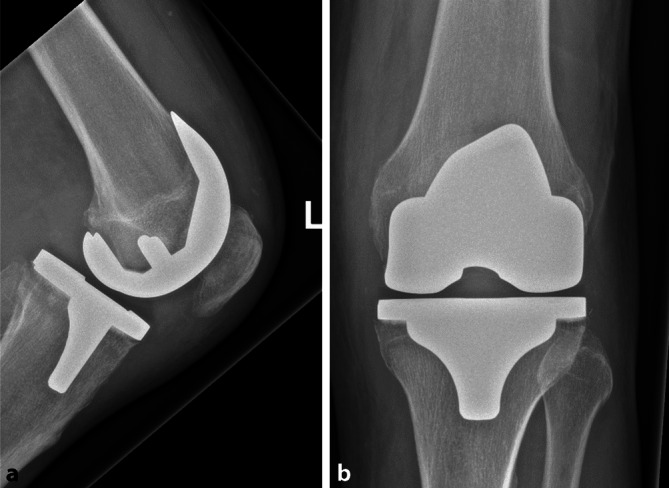

Für Protheseninfektionen gab es bisher verschiedene Klassifikationssysteme, die jedoch die Komplexität, welche die Erkrankung mit sich bringt, nicht vollumfänglich abbilden [24, 27]. Daher wurde eine Klassifikation für Protheseninfektionen entsprechend der TNM-Klassifikation in der Onkologie entwickelt, die verschiedene Dimensionen, die für die Behandlung von periprothetischen Infektionen wichtig sind, beinhaltet (Abb. 1; [1, 2]). Die Buchstaben TNM tauchen in der PJI-TNM-Klassifikation analog zur onkologischen Klassifikation auf, stehen jedoch für Faktoren, die bei periprothetischen Infektionen von Relevanz sind. „T“ steht für „tissue and implant“. Hierunter werden die Stabilität des Implantates, der Weichteilstatus und die Art der Prothese (Standard- vs. Revisionsimplantat) berücksichtigt. Unter „N“ werden die Erreger und die Reife des Biofilms berücksichtigt. „M“ beinhaltet die Nebenerkrankungen der Patienten, während ein vorangestelltes „r“ für ein Rezidiv einer Protheseninfektion verwendet wird. Zudem wird das betroffene Gelenk mit dem Namen der PJI-TNM Klassifikation vorangestellt.

Ziel dieser Arbeit war es, anhand einer Fallserie von Patienten zu demonstrieren, dass die neue PJI-TNM-Klassifikation eine bisher in der Klassifizierung von periprothetischen Infektionen unzureichend beachtete Heterogenität detailliert wiedergeben und somit als Grundlage für eine gezielte Therapieplanung und wissenschaftliche Auswertung von Therapiealternativen dienen kann.

## Untersuchungsmethoden

Insgesamt wurden 20 Patienten mit periprothetischer Infektion, die in der Klinik und Poliklinik für Unfallchirurgie des Universitätsklinikums Regensburg zwischen dem 01.03.2011 und 31.12.2019 behandelt wurden, zufällig über eine Diagnoseliste, die anhand des Diagnosecodes T84.5 erstellt wurde, ausgewählt. Anhand der Aktenlage und der EDV-basierten Daten wurde die PJI-TNM-Klassifikation eines jeden Patienten bestimmt. Die Klassifikation wurde anhand folgender Empfehlung (Abb. 1) abgeleitet:

- *T* („tissue and implant“) beinhaltet die Situation des Implantates und der periimplantären Weichteile. T0 kennzeichnet eine Situation mit einem nicht gelockerten Implantat und gesundem Weichteilmantel. In T1-Situationen ist das Implantat gelockert und der Weichteilmantel intakt. T2 beschreibt Situationen mit Weichteildefekten, die einer plastischen Deckung bedürfen, resp. bei denen kein primärer Wundverschluss zu erzielen ist, unabhängig vom Sitz des Implantates. Zusätzlich zu der Nummerierung werden noch Buchstaben für das Vorliegen eines Standardimplantates (a) oder einer Revisionsendoprothese (b) vergeben.
- *N* („non-eucaryotic cells and fungi“): „N“ steht für den ursächlichen Erreger und beinhaltet zudem die Dauer des bestehenden Infektes zur Abschätzung der Reife des Biofilms. N0 steht für Infektionen, bei denen kein reifer Biofilm vorhanden ist und beinhaltet somit die frühere Bezeichnung eines Akutinfektes. Hier wird zwischen direkt postoperativ (N0a) oder spät hämatogener (N0b) periprothetischer Infektion unterschieden. Mit N1 werden Infektionen mit etabliertem Biofilm bezeichnet, welche der früheren Bezeichnung eines chronischen Protheseninfektes gleichkommen und zudem mit gängigen Therapiealgorithmen gut zu therapieren sind. N1a wird verwendet, wenn ein Erreger vorliegt, welcher nicht gegen Rifampicin oder Ciprofloxacin resistent ist („non-difficult-to-treat“). Als N1b werden chronische Infektionen bezeichnet, bei welchen kein Erreger nachgewiesen werden konnte, bei denen jedoch eine periprothetische Infektion gesichert wurde [16]. Chronische Infekte, welche sich schwieriger behandeln lassen, werden unter N2 subsummiert und abhängig von den nachgewiesenen Erregern spezifiziert: N2a = chronischer Infekt mit einem Rifampicin- oder Ciprofloxacin-resistenten Erreger, N2b = polymikrobieller chronischer Infekt, N2c = chronischer Infekt mit Pilzen.
- *M* („morbidities“): Unter „M“ werden Vorerkrankungen des Patienten berücksichtigt, die für den Therapieerfolg von entscheidender Bedeutung sind [13]. Der Charlson-Komorbiditätsindex wird hierzu in seiner ursprünglichen Version verwendet ([8]; Tab. 1). M0 wird für Patienten mit einem Charlson-Index von 0–1 verwendet, M1 für Patienten mit einem Wert von 2–3, M2 für einen Score-Index von 4–5. M3 sollte bei Patienten angewandt werden, welche nicht operiert werden können bzw. sollten. Unter M3a wird der Wille des Patienten (Ablehnen der Operation) berücksichtigt, M3b bezeichnet Patienten die nicht von einer Operation hinsichtlich Mobilisation etc. profitieren, diese jedoch überleben würden. M3c-Patienten sollten nicht operiert werden, da das Überleben der Operation aus ärztlicher Erfahrung nicht gegeben wäre.
- *r* („reinfection“): Neben der TNM-Einteilung wird für Infektrezidive ein „r“ vor die dargestellte Einteilung gesetzt, um die deutlich erschwerte Therapie und das damit verschlechterte Outcome von erstmalig aufgetretenen periprothetischen Infektionen abzugrenzen.

**Table 1:** 

Erkrankung	Bewertung
Herzinfarkt^a^	1
Herzinsuffizienz^b^	1
Periphere arterielle Verschlusskrankheit^c^	1
Zerebrovaskuläre Erkrankungen^d^	1
Demenz^e^	1
Chronische Lungenerkrankung^f^	1
Kollagenose^g^	1
Ulkuskrankheit^h^	1
Leichte Lebererkrankung^i^	1
Diabetes mellitus (ohne Endorganschäden)^j^	1
Hemiplegie	2
Mäßig schwere und schwere Nierenerkrankung^k^	2
Diabetes mellitus (mit Endorganschäden)^l^	2
Tumorerkrankung^m^	2
Leukämie^n^	2
Lymphom^o^	2
Mäßig schwere und schwere Lebererkrankung^p^	3
Metastasierter solider Tumor	6
Aids	6

^a^Patienten mit Zustand nach Myokardinfarkt

^b^Patienten mit durch Anstrengung induzierter Dyspnoe, nächtlicher Dyspnoe, Angina pectoris, Z. n. koronarem Bypass

^c^Patienten mit Claudicatio intermittens, nach peripherer Bypassversorgung, mit akutem arteriellem Verschluss oder Gangrän sowie nicht versorgtem abdominellem oder thorakalem Aortenaneurysma größer als 6 cm

^d^Patienten mit transitorischer ischämischer Attacke oder Apoplex ohne oder nur mit geringfügigen Residuen

^e^Patienten mit chronischem kognitivem Defizit

^f^Patienten mit pulmonal bedingter Dyspnoe bei leichter oder mäßig schwerer Belastung ohne Therapie oder Patienten mit anfallsweiser Dyspnoe (Asthma)

^g^Polymyalgia rheumatica, Lupus erythematodes, schwere rheumatoide Arthritis, Polymyositis

^h^Patienten, die bereits einmal wegen Ulzera behandelt wurden

^i^Leberzirrhose ohne portale Hypertonie

^j^Patienten mit Diabetes mellitus und medikamentöser Therapie

^k^Dialysepflichtigkeit oder Kreatinin >3 mg/dl

^l^Retinopathie, Neuropathie oder Nephropathie

^m^Sämtliche solide Tumoren ohne Metastasennachweis innerhalb der letzten 5 Jahre

^n^Akute und chronische Leukosen

^o^Hodgkin- und Non-Hodgkin-Lymphome, multiples Myelom

^p^Leberzirrhose mit portaler Hypertonie ohne stattgehabte Blutung und Patienten mit Varizenblutung in der Anamnese

Der PJI-TNM-Klassifikation wird der Name des betroffenen Gelenkes vorangestellt, sodass zusätzlich zu T, N, M und r auch die Lokalisation des Infektes in die Klassifikation mit einfließt.

Für die Klassifizierung wurden neben den Aufnahmebriefen, Röntgenbildern, mikrobiologische Befunde, Laborwerte und Operationsberichte der Patienten gesichtet. Zur Beurteilung der Implantatstabilität wurde der klinische intraoperative Befund verwendet. Als Standardprothesen wurden die von verschiedenen Herstellern implantierten Gelenkkomponenten bewertet, die nicht für eine zusätzliche knöcherne Defektrekonstruktion nötig waren. Der Weichteilstatus wurde anhand des im Aufnahmebefund und im Operationsbericht notierten klinischen Bildes beurteilt. Die mikrobiologische Diagnostik umfasst hierbei die 14-tägige Kultur von Gelenkpunktaten, Gewebeproben und Sonikaten explantierter Prothesenkomponenten. Neben den Kulturergebnissen wurden die Ergebnisse der routinemäßig durchgeführten Bakterien-PCR („polymerase chain reaction“; 16S rDNA) mitberücksichtigt. Die Bewertung des Charlson-Komorbiditätsindexes erfolgte anhand der Tab. 1 [6].

Die Auswertung der Patientendaten wurde im Vorfeld durch die zuständige Ethikkommission der Universität Regensburg genehmigt (EA: 20-1681-104).

## Ergebnisse

Insgesamt wurden 20 Fälle mit 12 männlichen und 8 weiblichen Patienten bei einem mittleren Alter von 72,2 Jahren (40–88 Jahre) analysiert.

Bei allen 20 Patienten konnte die neue Klassifikation erfolgreich angewendet werden und die zugrunde liegende Protheseninfektion mit dem PJI-TNM-Schema klassifiziert werden. Es wurden 13 Hüftprotheseninfektionen, 6 Knieprotheseninfektionen und eine Schulterprotheseninfektion evaluiert. Bei 3 Fällen handelte es sich um eine Reinfektion (r). In 12 Fällen fand sich kein Lockerungszeichen der einliegenden Prothese mit guten Weichteilverhältnissen (T0), wobei davon 3 Prothesen Revisionsimplantate waren (T0b). Insgesamt fanden sich 7 Infektionen mit unreifem Biofilm (N0). Hierbei handelte es sich um eine postoperative Infektion und eine direkte Kontamination bei Décollementverletzung (N0a [akuter Infekt]) und fünf späthämatogene Infekte (N0b). 10 Protheseninfektionen wurden mit N1 klassifiziert, was einer Infektion mit reifem Biofilm und einem nachgewiesenen „non-difficult-to-treat“-Keim (N1a) oder keinem Keimnachweis (N1b) entspricht. In den restlichen 3 Fällen wurde der N‑Status mit N2 klassifiziert. Es fand sich zweimal ein „difficult-to-treat“-Erreger und einmal eine polymikrobielle Infektion. Hinsichtlich der Morbidität wurden 9 Patienten behandelt, welche nur geringgradig vorerkrankt waren (M0), 7 Patienten waren mäßig vorerkrankt (M1) und 3 Patienten waren schwer vorerkrankt (M2), ein Patient lehnte die chirurgische Behandlung ab (M3a) (Tab. 2).

**Table 2:** 

Patient	Geschlecht	Alter	Gelenk	Reinfektion (Protheseninfektion im selben Gelenk)	Lockerung	Weichteildefekt/Fistel	Implantatkategorie (S: Standard‑/Revisionsimplantat)	Einliegedauer der Prothese (J = Jahre, M = Monate)	Dauer von Symptom bis Operation (Monate)	Nachgewiesene Keime	Difficult-to-treat Erreger (Resistenz gegen Rifampicin [R], Ciprofloxacin [C], 0 = keine)	Relevante Vorerkrankungen nach CCI	CCI-Index	PJI-TNM-Klassifikation
#1	M	65	Hüfte links	Ja	Nein	Nein	R	7J + 3M	48	*Streptococcus agalactiae, Proteus vulgaris*	0	Herzinsuffizienz, Niereninsuffizienz Stadium IV, PAVK, Pat. lehnt operative Therapie ab	4	Hüfte-PJI-TNM rT0bN2bM3a
#2	W	84	Hüfte links	Nein	Nein	Nein	R	1M	0,5	*Candida krusei*	0	Herzinsuffizienz, Demenz, Depression, Niereninsuffizienz Stadium III	5	Hüfte-PJI-TNM T0bN0aM2
#3	W	87	Knie rechts	Nein	Nein	Nein	S	11J + 2M	0,25	Kein Keimnachweis	0	Polyzythämia vera	2	Knie-PJI-TNM T0aN0bM1
#4	M	83	Hüfte links	Nein	Nein	Nein	S	16J + 10M	0,25	*Enterococcus faecalis*	0	COPD, PAVK	2	Hüfte-PJI-TNM T0aN0bM1
#5	M	74	Hüfte links	Ja	Ja	Ja	R	3M	2,5	*Staphylococcus epidermidis*	R	Herzinsuffizienz Demenz, Diabetes Mellitus Typ 2	3	Hüfte-PJI-TNM rT2bN2aM1
#6	M	75	Knie links	Nein	Nein	Nein	S	6M	6	*Staphylococcus epidermidis*	0	Keine	0	Knie-PJI-TNM T0aN1aM0
#7	W	80	Knie rechts	Nein	Ja	Nein	S	6J	72	*Pseudomonas aeruginosa*	0	Herzinsuffizienz, COPD, Rheumatoide Arthritis, Niereninsuffizienz Stadium III	5	Knie-PJI-TNM T1aN1aM2
#8	W	88	Hüfte rechts	Nein	Nein	Nein	S	4J + 5M	0,25	MSSA	0	Z. n. Mammakarzinom (<5 Jahre seit Erstdiagnose)	2	Hüfte-PJI-TNM T0aN0bM1
#9	M	40	Totales Femur rechts	Ja	Nein	Nein	R	3J + 3M	71	*Staphylococcus haemolyticus*	R, C	Z. n. Ewing-Karzinom (>5 Jahre seit Erstdiagnose)	0	Hüfte-PJI-TNM rT0bN2aM0
#10	W	87	Knie rechts	Nein	Ja	Nein	S	3J + 9M	36	Koagulase-negative Staphylokokken	0	Herzinsuffizienz, Niereninsuffizienz Stadium IV	3	Knie-PJI-TNM T1aN1aM1
#11	W	63	Hüfte links	Nein	Ja	Nein	S	1J + 5M	17	*Proteus mirabilis*	0	Keine	0	Hüfte-PJI-TNM T1aN1aM0
#12	M	61	Schulter rechts	Nein	Nein	Nein	S	1J + 7M	6	*Cutibacterium acnes*	0	Keine	0	Schulter-PJI-TNM T0aN1aM0
#13	M	67	Knie links	Nein	Nein	Ja	S	2J + 3M	0 (Trauma mit Weichteildefekt)	*Enterococcus faecium, Aeromonas bestiarum*	0	Demenz, Niereninsuffizienz Stadium III	2	Knie-PJI-TNM T2aN0aM1
#14	M	83	Knie links	Nein	Nein	Nein	S	1J	0,25	*Staphylococcus epidermidis*	0	Keine	0	Knie-PJI-TNM T0aN0bM0
#15	W	84	Hüfte links	Nein	Nein	Nein	S	1J + 2M	6	*Pseudomonas aeruginosa*	0	Z. n. apoplektischem Insult, Z. n. Myokardinfarkt	2	Hüfte-PJI-TNM T0aN1aM1
#16	M	74	Hüfte links	Nein	Ja	Nein	R	13J + 3M	7	*Staphylococcus lugdunensis*	0	Z. n. Prostatakarzinom (>5 Jahre seit Erstdiagnose)	0	Hüfte-PJI-TNM T1bN1aM0
#17	M	52	Hüfte rechts	Nein	Ja	Nein	S	6M	3	*Staphylococcus epidermidis*	0	Keine	0	Hüfte-PJI-TNM T1aN1aM0
#18	M	55	Hüfte links	Nein	Nein	Nein	S	22J + 8M	0,5	MSSA	0	Keine	0	Hüfte-PJI-TNM T0aN0bM0
#19	W	76	Hüfte rechts	Nein	Ja	Nein	S	10 M	1	MSSA	0	Keine	0	Hüfte-PJI-TNM T1aN1aM0
#20	M	66	Hüfte links	Nein	Nein	Nein	S	18 J + 9 M	4	*Proteus mirabilis*	0	Herzinsuffizienz, Diabetes Mellitus Typ II, Niereninsuffizienz II, COPD, Gastritis	5	Hüfte-PJI-TNM T0aN1aM2

*CCI* Charlson-Komorbiditätsindex, *COPD* „chronic obstructive pulmonary disease“, *MSSA* Methicillin-sensitiver *Staphylococcus aureus*, *PAVK* periphere arterielle Verschlusskrankheit, *PJI* „periprosthetic joint infections“, *TNM* „tissue and implants, non-eucaryotic cells and fungi, morbidity“

Insgesamt zeigte sich eine deutliche Heterogenität der Fälle im klinischen Gesamterscheinungsbild, die sich dann ebenfalls in einer sehr heterogenen PJI-TNM-Klassifikation der untersuchten Protheseninfektionen ausdrückte. Die PJI-TNM-Klassifikation konnte daher die unterschiedlichen Protheseninfektionskonstellationen gut zum Ausdruck bringen, wie folgende Falldarstellungen beispielhaft verdeutlichen.

### Beispiel 1

„Chronische“ Kniegelenkprotheseninfektion 6 Monate nach Implantation mit Rifampicin-sensiblem *Staphylococcus epidermidis*. Regelrechter Weichteilstatus ohne Weichteildefekt, fester bikondylärer Oberflächenersatz, keine bekannten Vorerkrankungen (Tab. 2, Patient 6).

Der 75-jährige Patient klagte über Schmerzen im linken Knie, welche seit Erstimplantation eines bikondylären Oberflächenersatzes vor 6 Monaten bestanden. Die Weichteilsituation war regelrecht, es bestand keine Fistel oder gar ein größerer Weichteildefekt. Die Verlaufsröntgenaufnahmen zeigten keine Lockerungszeichen (Abb. 2). Somit lag hier bei nicht gelockertem Standardimplantat ohne Weichteilproblematik ein „T0a“-Status vor.

Im Punktat des Kniegelenkes wurde eine Gesamtzellzahl von 4500 Zellen/µl bei einem Granulozytenanteil von 92 % festgestellt. Die mikrobiologische Diagnostik des Punktates ergab den Nachweis eines *Staphylococcus epidermidis*, mit Rifampicin-Empfindlichkeit, was bei chronischem Verlauf und reifer Biofilmbildung einer „N1a“-Situation entspricht.

Vorerkrankungen bestanden keine, somit war das „M“-Kriterium mit „M0“ anzusetzen. Insgesamt wurde diese Situation somit als Knie-PJI T0aN1aM0 klassifiziert. Die Therapie bestand aus einem einzeitigen Knieprothesenwechsel.

### Beispiel 2

**Figure 3:**
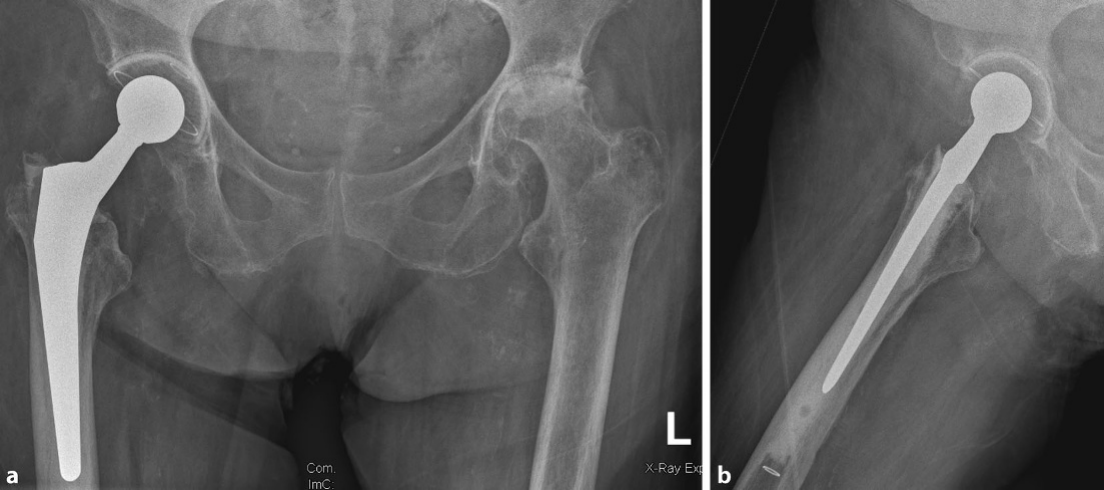

„Akute, spät hämatogene“ Hüftprotheseninfektion mit akuten Hüftschmerzen seit einer Woche durch Rifampicin-sensiblen *Staphylococcus aureus*. Regelrechte Weichteilverhältnisse und fester Implantatsitz einer Standardhüftprothese. Erstdiagnose Mammakarzinom vor 3 Jahren (Tab. 2 Patientin 8): Die 88-jährige Patientin klagte über akut aufgetretene Schmerzen seit 7 Tagen in der rechten Hüfte bei zuvor beschwerdefreiem Gelenk nach Erstimplantation einer zementierten Polyethylen-Pfanne und eines Standardhüftschaftes vor 4,5 Jahren. Aufgrund eines vor 3 Wochen behandelten Harnweginfekts war von einer „akuten Spätinfektion“ ausgegangen worden. Die Weichteile zeigten keine Auffälligkeit, die Röntgenbilder zeigten keine Lockerung des Implantates (Abb. 3). Somit war das „T“-Kriterium mit „T0a“ zu beurteilen.

Im putriden Punktat des Hüftgelenkes ließ sich ein * Staphylococcus aureus* mit Rifampicin-Empfindlichkeit nachweisen, dessen Biofilmbildung aufgrund der erst seit 7 Tagen bestehenden Beschwerden als „unreif“ und die Situation somit als „N0b“ definiert wurde.

An Vorerkrankungen war ein vor 3 Jahren diagnostiziertes Mammakarzinom und eine arterielle Hypertonie bekannt, was nach Charlson-Komorbiditätsindex einem Wert von 2 entsprach und somit als „M1“ klassifiziert wurde.

Insgesamt wurde die Infektion als Hüfte-PJI T0aN0bM1 klassifiziert. Die Patientin wurde implantaterhaltend durch Debridement, Spülung und Wechsel des Hüftkopfes sowie einer anschließenden Antibiotikatherapie mit Rifampicin und Flucloxacillin für 6 Wochen behandelt.

### Beispiel 3

**Figure 4:**
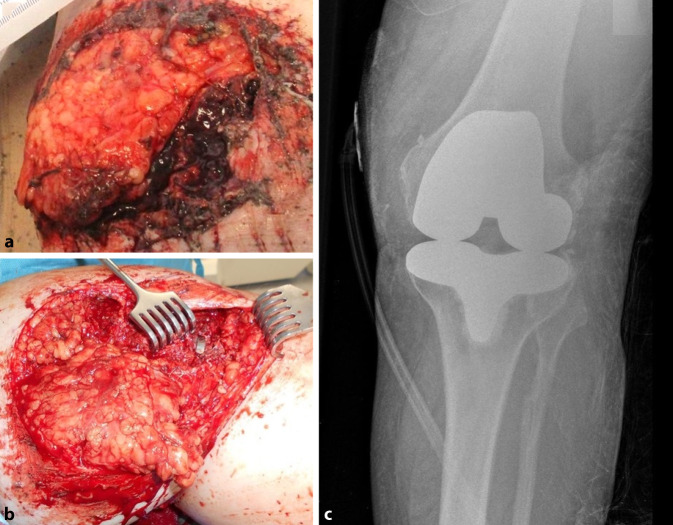

Traumatischer Weichteildefekt des linken Knies nach Überrolltrauma durch einen Traktor im Rahmen eines berufsgenossenschaftlich versicherten Unfalls bei einem 67-jährigen Patienten (Tab. 2, Patient 13). Fester Implantatsitz des bikondylären Oberflächenersatzes. Nachweis von *Enterococcus faecium* und * Aeromonas bestiarum* nach Debridement. Der Patient erlitt ein Überrolltrauma durch einen Traktor auf dem eigenen landwirtschaftlichen Hof. Hierbei kam es neben einem schweren Schädelhirntrauma, Thoraxtrauma und Abdominaltrauma zu einer tiefen Décollementverletzung am linken medialen Kniegelenk mit freiliegender Standard-Knie-TEP (Abb. 4). Daher wurde das „T“-Kriterium mit „T2a“ beurteilt. Nach dem chirurgischen Debridement direkt nach Übernahme des Patienten aus dem Schockraum konnten *Enterococcus faecium* und *Aeromonas bestiarum* mikrobiologisch nachgewiesen werden, was auf die polymikrobielle Kontamination durch die Defektwunde zurückzuführen war. Daher war das „N“-Kriterium bei noch nicht reifem Biofilm als „N0a“ zu werten. Der Patient litt bereits an einem beginnenden demenziellen Syndrom. Ferner bestand eine Niereninsuffizienz III°, sodass dies bei einem Charlson-Komorbiditätsscore von 3 einem „M1“ entsprach. Diese Knieprotheseninfektion wurde als Knie-PJI T2aN0aM1 klassifiziert. Die Knieprothese wurde trotz noch festem Sitz aufgrund des ausgeprägten Weichteildefektes mit freiliegender Prothese bei starker Verunreinigung im Rahmen eines geplanten zweizeitigen Wechsels ausgebaut, ein temporärer Gentamicin-beladener Polymethylmethacrylat-Spacer eingelegt und der Defekt plastisch-chirurgisch mit einer medialen Gastroknemiuslappenplastik verschlossen. Nach Beruhigung der Weichteilsituation wurde die Reimplantation einer Knie-TEP geplant.

## Diskussion

Aus klinischer und wissenschaftlicher Perspektive besteht dringlicher Bedarf für eine verbesserte Klassifikation für Protheseninfektionen. Die lokale Situation der infizierten Prothese, der Prothesentyp, die Erreger und dessen Antibiotikaempfindlichkeiten sowie die Dauer der Infektion, die Komorbiditäten und der Wille bzw. die Erwartungshaltung des Patienten, genauso wie das eventuelle Vorliegen eines Rezidivinfektes, sind entscheidende Faktoren, die für eine bessere Klassifikation von Protheseninfektionen berücksichtigt werden müssen. Dies ist notwendig, um daraus relevante Therapieempfehlungen abzuleiten und diese für wissenschaftliche Studien besser vergleichbar zu machen. Wie die vorgestellte Fallserie zeigt, ist der Vorteil der vorgeschlagenen PJI-TNM-Klassifikation, dass diese eine Vielzahl von relevanten Parametern bei Protheseninfektionen beinhaltet und dadurch die bestehende Heterogenität der Erkrankung adäquat abgebildet werden kann. Somit ist anzunehmen, dass sich, ähnlich wie in der Onkologie, Therapieempfehlungen durch eine PJI-TNM-Klassifikation besser ableiten lassen, als bei Verwendung einer Klassifikation, die lediglich zwischen einer akuten oder chronischen Protheseninfektion unterscheidet.

„T“ steht für den Tumor in der onkologischen TNM-Klassifikation. In der PJI-TNM-Klassifikation wird durch „T“ das Implantat mit dem umgebenden Gewebe („tissue“) erfasst. „N“ wird benutzt, um die mikrobiologischen Befunde und deren Relevanz in eine Klassifikation einfließen zu lassen. Die Autoren empfehlen diesbezüglich, sich dabei auf die Ausreifung des Biofilms zu beschränken, um so die genaue zeitliche Abgrenzung (akute vs. chronische Infektion resp. frühe vs. späte Infektion) – welche in der Literatur unterschiedlich angegeben wird und nicht sicher beziffert werden kann – zu umgehen [5, 21]. Zudem beinhaltet die Beachtung der Biofilmreife in einer Klassifikation die zusätzliche Therapieoption eines Implantaterhaltes (DAIR = „debridement, antibiotics, implant retention“) bei späthämatogenen Infektionen. Diese detailliertere Betrachtung in der PJI-TNM-Klassifikation gegenüber den einfachen dualistischen Klassifikationen, die nur die Infektionsdauer berücksichtigen, kann für die Patienten von deutlichem Vorteil sein. Die Morbidität („M“) der Patienten ist für die Behandlung, die Prognose der Patienten und zum Vergleich verschiedener Patientenkohorten in klinischen Studien von herausragender Bedeutung [10]. Bei der Klassifizierung der Osteomyelitis wurde diese Bedeutung bereits durch Georg Cierny erkannt, sodass er die Morbidität mit in die Klassifikation der Osteomyelitis hat einfließen lassen [9]. Bei periprothetischen Infektionen erfasst die McPherson-Klassifikation ähnlich zur Cierny-Mader-Klassifikation neben dem Infektionstyp nach Dauer der Infektion auch relevante lokale und systemische Faktoren [22]. Die PJI-TNM-Klassifikation berücksichtigt die Morbidität der Patienten unter Verwendung des Charlson-Komorbiditätsindexes, welcher eine zuverlässige Einschätzung der Risiken durch Alter und Nebenerkrankungen zulässt [6, 7].

Eine der Schwächen der PJI-TNM-Klassifikation ist die mögliche Verwechslung mit der bereits etablierten Tumorklassifikation, welche durch das Präfix PJI vermieden werden soll. Indes kann die PJI-TNM-Klassifikation keine Antwort auf die Frage geben, ob ein Biofilm reif oder unreif ist, solange es keine besseren diagnostischen Methoden als die derzeitigen gibt. Die weitere Differenzierung innerhalb einzelner Buchstaben (0 bis zu 3, zusätzlich a und b) lässt diese Klassifikation als kompliziert erscheinen, insbesondere gegenüber der einfachen und „landläufigen“ Unterscheidung zwischen akuter und chronischer bzw. Früh- und Spätinfektion. Nicht alle Kriterien, die bei der Diagnostik einer periprothetischen Infektion von entscheidender Bedeutung sind, fließen zudem allesamt in die PJI-TNM-Klassifikation ein [23]. Beispielsweise wird die differenzierte histopathologischen Diagnostik, wie sie von Krenn und Kollegen beschrieben wurde und ständig weiterentwickelt wird, nicht berücksichtigt [18, 19]. Die Autoren sind der Meinung, dass sich zunächst auf direkte therapeutische Konsequenzen, wie sie unter T, N und M subsummiert sind, beschränkt werden sollte, um einen guten Kompromiss zwischen Komplexität und Akzeptanz resp. Anwendung in der klinischen Praxis zu erzielen. Wertvolle histopathologische Diagnostik, die zwischen aseptischen, septischen Lockerungen und sogar seltenen malignen Prozessen, wie Lymphomen um einliegende Endoprothesen, unterscheiden kann, könnte in einem zweiten Schritt analog zur onkologischen TNM-Klassifikation als „Grading“ zur Geltung kommen [17].

Die Autoren sind der Meinung, dass die Komplexität der PJI-TNM-Klassifikation der Komplexität der Protheseninfektions-„Erkrankung“ mit all ihren Facetten, wie der lokalen Situation der Weichteile, dem Sitz und der Art des Implantates, den Erregern und der Dauer der Infektion, der Morbidität der Patienten und dem Vorhandensein einer Reinfektion, nachkommt. Die Aufführung der zufällig ausgewählten klinischen Fälle mit den darin schon absehbaren Unterschieden in den einzelnen Unterpunkten der Klassifikation, zeigt die Notwendigkeit einer differenzierteren Betrachtung des Krankheitsbildes, welche nun mit der PJI-TNM-Klassifikation möglich ist.

Als nächster Schritt ist die Validierung der PJI-TNM-Klassifikation hinsichtlich ihrer Anwendbarkeit im klinischen Alltag nötig, bevor in weiteren Untersuchungen auf Behandlungsergebnisse in Abhängigkeit von der Klassifikation und Therapie eingegangen werden kann. Ähnlich zur Onkologie soll bei periprothetischen Infektionen die PJI-TNM-Klassifikation in absehbarer Zeit helfen, eine geeignete Therapieempfehlung ableiten zu können.

## Fazit für die Praxis

- Die aus der Onkologie stammenden Prinzipien der TNM-Klassifikation lassen sich auch bei periprothetischen Infektionen anwenden.
- Schon bei einer geringen Fallzahl ist die Heterogenität, die Protheseninfektionen ausmacht, deutlich erkennbar.
- Da davon ausgegangen werden muss, dass diese Heterogenität entscheidenden Einfluss auf den Therapieerfolg der jeweiligen Therapieentscheidung hat, sollte in weiteren Studien die PJI-TNM-Klassifikation (PJI: „periprosthetic joint infections“, TNM: „tissue and implants, non-eucaryotic cells and fungi, morbidity“) validiert und auf deren klinische Relevanz hin untersucht werden.

## Abkürzungen

CCI: Charlson-Komorbiditätsindex
COPD: „Chronic obstructive pulmonary disease“
DAIR: „Debridement, antibiotics, implant retention“
MSSA: Methicillin-sensitiver *Staphylococcus aureus*
PAVK: Periphere arterielle Verschlusskrankheit
PCR: „Polymerase chain reaction“
PJI: „Periprosthetic joint infections“
TEP: Totalendoprothese
