# Erratum zu: PJI-TNM als neues Klassifikationssystem für Endoprotheseninfektionen: Eine Evaluation von 20 Fällen

**DOI:** 10.1007/s00132-021-04123-7

**Published:** 2021-06-22

**Authors:** Markus Rupp, Maximilian Kerschbaum, Viola Freigang, Susanne Bärtl, Florian Baumann, Andrej Trampuz, Volker Alt

**Affiliations:** 1 Klinik und Poliklinik für Unfallchirurgie, Universitätsklinikum Regensburg (UKR), Franz-Josef-Strauß-Allee 11, 93053 Regensburg, Deutschland; 2 Charité – Universitätsmedizin Berlin und Center for Musculoskeletal Surgery (CMSC), Berlin, Deutschland

**Erratum zu:**

**Orthopäde 2020**

10.1007/s00132-020-03933-5

Der Artikel „PJI-TNM als neues Klassifikationssystem für Endoprotheseninfektionen. Eine Evaluation von 20 Fällen“ von Markus Rupp, Maximilian Kerschbaum, Viola Freigang, Susanne Bärtl, Florian Baumann, Andrej Trampuz und Volker Alt wurde ursprünglich Online First ohne „Open Access“ auf der Internetplattform des Verlags publiziert. Nach der Veröffentlichung in Bd. 50 Heft 3 pp. 198–206 hatten sich die Autoren für eine „Open Access“-Veröffentlichung entschieden. Das Urheberrecht des Artikels wurde deshalb in © The Author(s) 2020 geändert.

